# Analysis of lncRNA UCA1‐related downstream pathways and molecules of cisplatin resistance in lung adenocarcinoma

**DOI:** 10.1002/jcla.23312

**Published:** 2020-04-06

**Authors:** Huixin Zhou, Qiang Shen, Jiali Fu, Feng Jiang, Liangxing Wang, Yumin Wang

**Affiliations:** ^1^ Department of Laboratory Medicine The First Affiliated Hospital of Wenzhou Medical University Wenzhou China; ^2^ Department of Respiratory The First Affiliated Hospital of Wenzhou Medical University Wenzhou China

**Keywords:** lung adenocarcinoma, signaling pathway, TXNIP, UCA1

## Abstract

**Background:**

To analyze the lncRNA UCA1‐related downstream pathways and molecules of cisplatin resistance in lung adenocarcinoma.

**Methods:**

We constructed overexpression and siRNA vectors targeting UCA1 and TXNIP and then used next‐generation sequencing to compare the UCA1 overexpression and negative control from A549 cell.

**Results:**

It shown that 647 upregulated mRNAs and 633 downregulated differentially expressed mRNAs‐related UCA1, and the top ten upregulated mRNAs were CPD, AC007192.1, TGOLN2, LGR4, TFPI, CYP1B1, TOMM6, HLA‐B, SLC35F6, and TOP2A, and top ten downregulated mRNAs were TXNIP, SESN2, STC2, HSPA1A, MMP10, CHAC1, DNAJB1, AC004922.1, ATF3, and GABARAPL1. We found TXNIP mRNA expression level was the most significantly downexpressed mRNA. TXNIP mRNA expression level of LAD tissues was clearly lower than the adjacent tissues. UCA1 expression level of cisplatin insensitive group was markedly higher than that of cisplatin‐sensitive group, while TXNIP mRNA expression level of cisplatin insensitive group was clearly lower than that of cisplatin‐sensitive group. Compared to the BEAS‐2B, TXNIP mRNA expression level cut down in A549 and A549/DDP cell and that of A549/DDP cell was lower than A549 cell. After UCA1 overexpression, TXNIP mRNA obviously decreased, while proliferation ability and IC50 of A549 heightened. After knocking down UCA1, TXNIP mRNA was significantly increased, while proliferation ability and IC50 of A549/DDP lowered. PPI analysis result showed that TXNIP could interact with multiple proteins such as TXN, DDIT4, and NLRP3.

**Conclusion:**

UCA1 promoted cisplatin resistance by downregulating TXNIP expression in LAD, and TXNIP could interact with multiple proteins. So, UCA1/TXNIP axis might affect cisplatin resistance in LAD.

## 1 INTRODUCTION

Lung adenocarcinoma (LAD) is one of the most common types of NSCLC, accounting for 40% of lung cancer. It is on the rise for LAD had the biological characteristics of early distant metastasis, and most patients are diagnosed at an advanced stage. Therefore, cisplatin‐based combination chemistry still played an important role in its comprehensive treatment program.^1^ With the wide application of cisplatin, it would inevitably cause tumor cells to develop resistance to it, so that the chemotherapy effect was significantly reduced.^2^ Studies had shown that 70%‐80% of patients can temporarily relieve the disease in the early stage of chemotherapy, but long‐term application would produce resistance to cisplatin, resulting in a recurrence rate of more than 60%, while drug resistance of recurrent lung cancer is significantly heightened, chemotherapy drug remission rate is less than 30%,^3^ the current chemotherapy rate of patients with advanced LAD is only 30%‐40%, and the 5‐year survival rate is less than 15%.^4^ According to the American Cancer Society survey, more than 90% of cancer patients' deaths are related to tumor drug resistance to varying degrees. Once cancer cells are resistant to cisplatin, they will be resistant to doxorubicin, vinblastine, fluorouracil, mitomycin, etc So, many first‐line chemotherapeutic drugs become multi‐drug resistance to cancer cells, which is particularly harmful.^5^

UCA1 was first confirmed to be highly expressed in bladder cancer tissues^6^ and shown to be highly expressed in other cancers. We had used high‐throughput lncRNA microarray technology to compare LAD cisplatin‐resistant A549/DDP cells and cisplatin‐sensitive A549 cells and obtained differential lncRNA expression profiles of LAD cisplatin‐resistant cell. Among them, we confirmed UCA1 was an upregulated lncRNA in these candidate lncRNAs by microarray and qPCR. Subsequently, our qPCR showed that the expression of UCA1 in LAD was significantly higher than that in adjacent tissues, which was consistent with the related reports,^7^, ^8^ indicating that UCA1 might play an important role in the development of LAD. It had been reported in the literature that UCA1 was involved in the cisplatin resistance mechanism of ovarian cancer and bladder cancer.^9^, ^10^ Therefore, we speculated that UCA1 might be a novel lncRNA molecule that plays an important role in LAD resistance to cisplatin. How would UCA1 regulate the cisplatin resistance mechanism of LAD through downstream signaling pathways and molecules? These issues deserved our in‐depth study, but no relevant literature reports had been reported so far. So, we used a high‐throughput next‐generation sequencing (NGS) to compare the UCA1 overexpression (A549 OE) and negative control from A549 cell (A549 OE NC) samples to analyze lncRNA UCA1‐related downstream pathways and molecules of cisplatin resistance in LAD.

## 2 MATERIALS AND METHODS

### 2.1 Human LAD tissue specimen

Human LAD tissue specimens were collected from the First Affiliated Hospital of Wenzhou Medical University from 2010 to 2015. Specimens were obtained through lung puncture biopsy, surgical resection, and metastatic lymph node biopsy and were strictly identified by the pathology department. All tissue specimens must meet the following conditions: Patients with primary LAD and clinical stage are IIIB to IV. First‐line chemotherapy was cisplatin 25 mg/m^2^ on the first 1‐3 days, combined with gemcitabine 1000 mg/m^2^ or paclitaxel 80 mg/m^2^ on days 1 and 8. The 21 days were one cycle, and each patient was treated for 3‐4 cycles. According to medical imaging examinations such as CT and MRI and detection of serum tumor markers and the RECIST standard, they were divided into "cisplatin‐sensitive group" (complete response + partial response) and "cisplatin insensitive group" (progression), and a total of sensitive groups were collected. There were 25 specimens and 32 insensitive specimens. Tissue specimens were stored in liquid nitrogen before use.

### 2.2 Cell culture of LAD cell line and cisplatin‐resistant strain

A549 cells and cisplatin‐resistant cell line A549/DDP were added with appropriate amount of RPMI1640 medium containing 10% calf serum and gently pipetted into a single‐cell suspension with a pipette, and the cell suspension was transferred to a cell culture flask with a pipette. The cells were cultured in a 5% CO_2_ incubator at 37°C, and A549/DDP cells were required to add cisplatin at a concentration of 2 μg/mL to maintain drug resistance. The medium was changed every 2‐3 days. The cell growth state was observed to be good, and the cell passage was performed at a density of 70%‐90%.

### 2.3 Lentivirus‐mediated overexpression vector construction and transfection

We constructed overexpression GV303 vector and siRNA GV248 vector targeting UCA1 and TXNIP (Gene Chem, Shanghai, China). Transfections were performed by seeding 2 × 10^5^ cells in 6‐well plate. After 24 hours, these mediums were replaced, and the cells (including A549 and A549/DDP) were incubated with the transfection complex according to the manufacturer's protocol. The multiplicity of infection values was as follows: A549 MOI = 10 and A549/DDP MOI = 10. The cells were infected with lentivirus for 72 hours, and overexpression efficiency was detected by qPCR. Puromycin screen test obtained the cell lines successfully transfected with these lentivirus‐mediated vectors. The study included A549 UCA1 overexpressing cell line (A549 UCA1 OE), and A549 was infected with lentivirus negative control LVCON145 vector (A549 UCA1 OE NC), A549/DDP TXNIP overexpressing cell line (A549/DDP TXNIP OE), and A549/DDP was infected with lentivirus negative control LVCON145 vector (A549/DDP TXNIP OE NC), A549/DDP UCA1 siRNA, A549/DDP was infected with lentivirus negative control LVCON077 vector (A549/DDP UCA1 siRNA NC), and A549 TXNIP siRNA and A549 were infected with lentivirus negative control LVCON145 vector (A549 TXNIP siRNA NC).

### 2.4 Cell proliferation assay

Cell Counting Kit‐8 (CCK‐8, Corning Corporation) was evaluated to cell proliferation assay comply with the manufacturer's standard operating procedure.^11^ In short, 3000 cells were resuspended and inoculated in 96‐well plates in the presence of 10% FBS for 1 week. On the second day, the cells were incubated with 10 μL CCK‐8 for 1 hour, and the absorbance was measured at 450 nm with TECAN (Germany) on 0, 24, 48, and 72 hours.

### 2.5 Cisplatin sensitivity test

The cell inoculation density was 2000/well. After 24 hours of cell attachment, 100 μL of complete medium (including cisplatin) was added to each well. The concentration of cisplatin was 1, 2, 4, 10, and 25 μg/mL, and only the zeroing hole of the medium and the control hole of the single‐cell suspension were set to zero. After 48 hours of culture, the complete medium containing 10% CCK8 was used instead of the medium and kept training for 45 minutes. The microplate reader detects the absorbance at 450 nm wavelength, cell viability % = (A plus‐A blank)/(A0 plus drug‐A blank) × 100%, using the SPSS18.0 software profit regression model to calculate the IC50 of the cell. Each group was provided with three sets of repeat holes.

### 2.6 Sequencing and data analysis

Through NanoDrop ND‐1000 concentration measurement, 1‐2 µg of total RNA was selected from each sample for the construction of an RNA sequencing library. The specific process is as follows. The total RNA samples were enriched with oligo dT (rRNA removal) treatment, and then, KAPA Stranded RNA‐Seq Library was selected. Prep Kit (Illumina) builds the library. The double‐stranded cDNA synthesis in the library construction process uses dUTP method in combination with subsequent high‐fidelity PCR polymerase to make the final RNA sequencing library has strand specificity. The constructed library was identified by Agilent 2100 Bioanalyzer Quality and library quantification by qPCR method. The mixed different sample libraries were sequenced using the Illumina NovaSeq 6000 sequencer, including NaOH alkaline denaturation single‐strand generation, Illumina flow cell in situ amplification, 150 double‐end cycle sequencing, and other steps.

Using Solexa pipeline version 1.8 (Off‐Line Base Caller software, version 1.8) software for image processing and base recognition. FastQC software evaluated the read quality of the reads after delinking (using cutadapt to 3′ and 5′ linkers). Through Hisat2 software match to reference base group. Used StringTie software to refer to the official database annotation information to estimate transcription abundance.^12^, ^13^ Used R software Ballgown^12^, ^13^ to perform FPKM^14^ calculations at the gene level and transcript level and calculated the expression differences between the gene level and transcript level, respectively. Differentially expressed genes. New genes/transcript predictions were assembled, merged, and compared with the official annotation information by StringTie for each sample separately. They were obtained through Ballgown calculations, and the new transcripts were predicted by CPAT.^15^, ^16^ We also used GO function significance enrichment analysis, pathway significance enrichment analysis, and other data mining analysis.

### 2.7 qPCR

Take 0.1 g of tissue, ground it thoroughly with liquid nitrogen (to powder) or discard the medium with 1‐5×10^7^ cells, and rinse with pre‐chilled PBS twice. Total RNA was extracted from cell or tissues samples using TRIzol reagent (Invitrogen life technologies) and then extracted into cDNA according to the manufacturer's instructions using Thermo Scientific RT Kit (Thermo Scientific). These different mRNA expressions were measured by qPCR on the ABI 7500 analyzer. The primers of these genes are shown in Table 1. PCR was carried out in a total volume of 20 μL, including 10 μL SYBR premix (2×), 2 μL cDNA template, 1 μL meaningful primer (10 mmol/L), 1 μL antisense primer (10 mmol/L), and 6 μL double distilled water. The qPCR reaction was set at an initial denaturation step of 10 minutes at 95°C; and 95°C (5 seconds), 60°C (30 seconds) in a total 40 cycles with a final extension step at 72°C for 5 minutes. All experiments were conducted in triplicate, and the median of each triplicate was used to calculate the relative mRNA concentration relative to β‐actin (ΔCt = Ct median mRNAs‐Ct median β‐actin) and 2^−ΔΔCt^ formula for the relative expression.^17^

**TABLE 1:** The primers of UCA1 and these candidate genes

Gene ID	Sense primer	Antisense primer	Product size (bp)
UCA1	ACGCTAACTGGCACCTTGTT	CTCCGGACTGCTTCAAGTGT	124
TGOLN2	CATCACAACAAGCGGAAGATCA	AGGAGGTGCAGGGAAAACATT	138
GR4	CGAATGGGCAGACCAGAACT	AGCTGACAGCACGCAGTTTA	136
TFPI	AGATGGTCCGAATGGTTTCCA	GCCGCATTCTTCCAACCATC	141
TOMM6	GGGGTGTAGCCAAGTAGTTCT	TCTGCGTGGACTCAAGGAAC	214
TXNIP	CAACTTGCTGCCCGACAAAA	CCAGAGGCCCCTGAGGAA	273
SESN2	CATCCAGGCCTTGCTGAAGA	GCCAAACACGAAGGAGGAGA	109
STC2	CCTGCAGAATACAGCGGAGA	AGCGTGGGCCTTACATTTCA	202
HSPA1A	AGCTGGAGCAGGTGTGTAAC	CAGCAATCTTGGAAAGGCCC	154
MMP10	GACAGAAGATGCATCAGGCAC	GGCGAGCTCTGTGAATGAGT	133
β‐actin	CCTGGCACCC AGCACAAT	GCTGATCCACATCTGCTGGAA	157

### 2.8 Statistical methods

Statistical analysis was performed for the comparison of two groups using Student's *t* test and one‐way ANOVA test for differences in variables between the groups from normal distribution data. The threshold value we used to designate differentially expressed mRNAs and mRNAs was a fold change of ≥2.0 or ≤0.5 (*P* < .05). Differences with *P* < .05 were considered statistically significant in both cases.

## 3 RESULTS

### 3.1 Construction of lentivirus‐mediated UCA1 overexpression and siRNA vector

**FIGURE 1:**
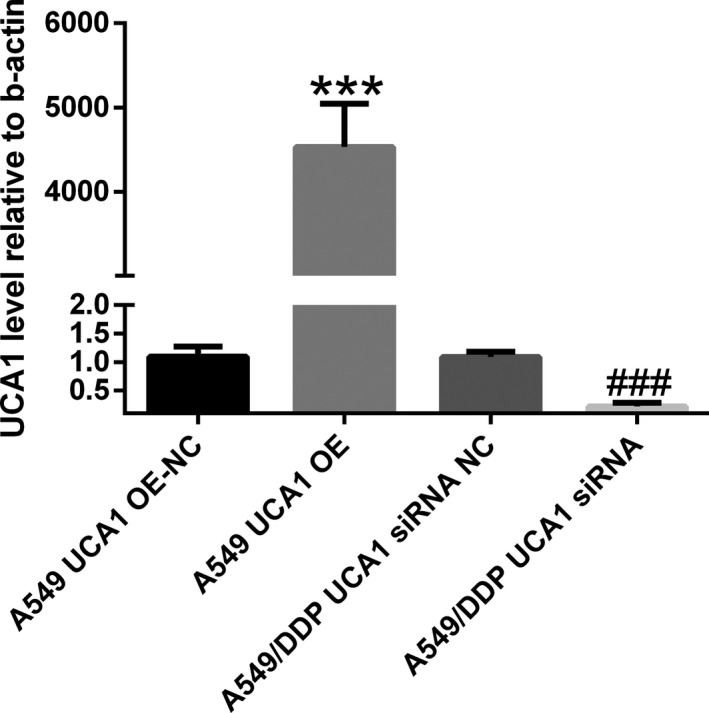
Construction of lentivirus‐mediated UCA1 overexpression and siRNA vector. The overexpression level of UCA1 in A549 UCA1 OE group (8013.33 ± 234.45) was significantly higher than that in the A549 UCA1 OE NC groups (*t* = 15.26, *P* = .0001, Figure 1). The overexpression level of UCA1 in A549 UCA1 siRNA group (8013.33 ± 234.45) was clearly lower than that in the A549 UCA1 siRNA NC groups (*t* = 13.45, *P* = .0002). ***P < .001, ###*P* < .001

The overexpression level of UCA1 in A549 UCA1 OE group (8013.33 ± 234.45) was significantly higher than that in the A549 UCA1 OE NC groups (*t* = 15.26, *P* = .0001, Figure 1). The overexpression level of UCA1 in A549 UCA1 siRNA group (8013.33 ± 234.45) was clearly lower than that in the A549 UCA1 siRNA NC groups (*t* = 13.45, *P* = .0002, Figure 1). These results showed that we had successfully established UCA1 overexpressing and siRNA cell lines.

### 3.2 Quality assurance of RNA and quality control results of mRNA sequencing

According to the OD of A260/A280 ratio should be close to 2.0 for pure RNA (ratios between 1.8 and 2.1 were acceptable). The OD A260/A230 ratio should be more than 1.8 and Table 2, so our RNA quantification was passed. Generally, Q30 ≥ 80% indicates that the sequencing quality is extremely high. Our findings showed that the results of Q30 were higher than 90%, and hinted sequencing quality was qualified (shown in Table 3).

**TABLE 2:** RNA quantification and quality assurance by NanoDrop ND‐1000

Sample ID	OD260/280 ratio	OD260/230 ratio	Conc. (ng/μL)	Volume (μL)	Quantity (ng)	QC purity
UCA1‐1	2.02	1.97	652.19	50	32 609.50	Pass
UCA1‐2	1.98	2.38	593.05	50	29 652.50	Pass
UCA1‐3	1.99	2.36	775.39	40	31 015.60	Pass
NC‐1	2.01	2.20	756.07	40	30 242.80	Pass
NC‐2	1.99	2.31	807.44	40	32 297.60	Pass
NC‐3	2.02	2.29	929.64	40	37 185.60	Pass

**TABLE 3:** The result of Q value statistics

Sample ID	Reads number	Total number of bases	Base number (*Q* ≥ 30)	Q30 (%)
NC‐1	50 289 784	7 543 467 600	6 932 617 011	91.90
NC‐2	43 592 066	6 538 809 900	5 973 874 183	91.36
NC‐3	57 044 504	8 556 675 600	7 868 420 997	91.96
UCA1‐1	42 537 012	6 380 551 800	5 857 299 554	91.80
UCA1‐2	46 690 666	7 003 599 900	6 469 682 313	92.38
UCA1‐3	36 903 756	5 535 563 400	5 042 348 811	91.09

### 3.3 Overview of mRNA profiles

**FIGURE 2:**
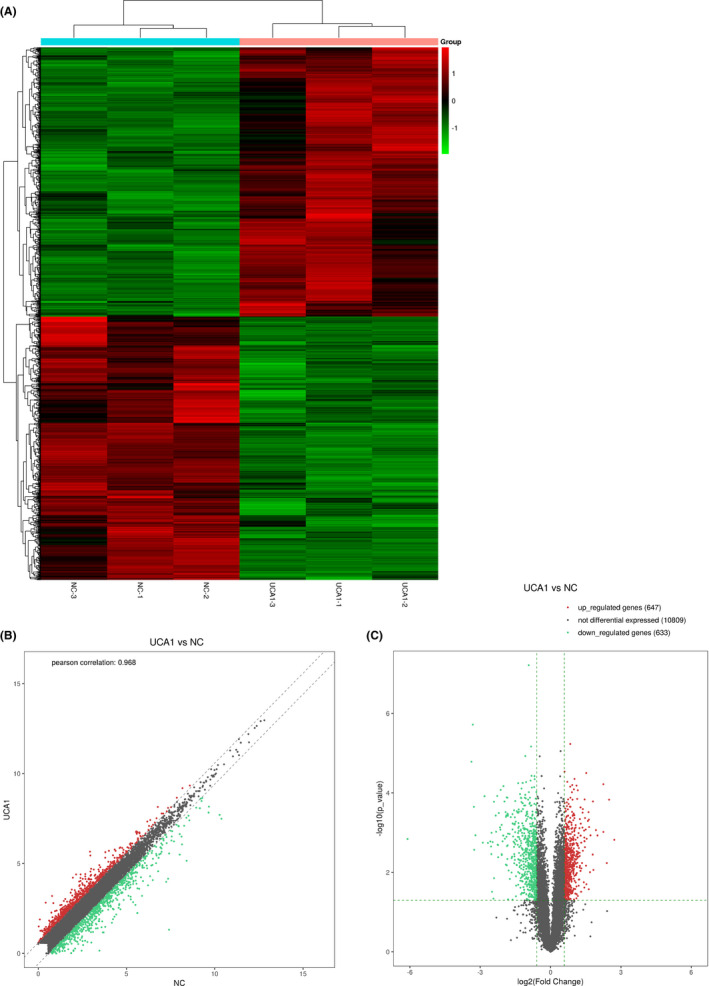
The different mRNAs profile comparison between the A549 UCA1 OE and A549 UCA1 OE NC samples. A, Cluster diagram of differentially expressed genes of mRNA profile *comparison* between the A549 UCA1 OE and A549 UCA1 OE NC samples. Each row represents a gene, and each column represents a sample. Red represents upregulated significantly differentially expressed genes, and green represents downregulated significantly differentially expressed genes. B, The scatter plot is used for assessing the mRNA expression variation between A549 UCA1 OE and A549 UCA1 OE NC samples and *r* = .968, so the overall distribution trend of the two sets of data is consistent. C, Volcano plot displayed the differentially expressed genes between different sample groups. The two vertical green lines were upregulated (right side > and downregulated (left side>), and the green parallel lines correspond to p‐value thresholds. Green dots represent significantly different downregulated genes, red dots represent significantly different upregulated genes, and gray dots represent non‐significantly different genes

The results showed that the expression profiles of mRNAs indicated that they were differentially expressed (fold change ≥ 2.0 or ≤0.5; *P* < .05) between A549 OE and A549 OE NC group. Among these, 647 mRNAs were found to be upregulated more than two‐fold in the A549 OE group compared to the A549 NC group, while 633 mRNAs were downregulated more than two‐fold (*P* < .05, Figure 2A‐C). The result showed that the top ten upregulated mRNAs were CPD, AC007192.1, TGOLN2, LGR4, TFPI, CYP1B1, TOMM6, HLA‐DMB, SLC35F6, and TOP2A, and top ten downregulated mRNAs were TXNIP, SESN2, STC2, HSPA1A, MMP10, CHAC1, DNAJB1, AC004922.1, ATF3, and GABARAPL1(shown in Table 4).

**TABLE 4:** Top 10 upregulated and downregulated mRNAs related to UCA1

Track_id	Gene_Name	Up or down	Fold_Change
ENSG00000108582.11_2	CPD	Up	3.50390
ENSG00000268173.3_4	AC007192.1	Up	3.48109
ENSG00000152291.13_2	TGOLN2	Up	3.25879
ENSG00000205213.13_2	LGR4	Up	3.20838
ENSG00000003436.15_3	TFPI	Up	3.12281
ENSG00000138061.11_3	CYP1B1	Up	3.10632
ENSG00000214736.7_3	TOMM6	Up	3.09472
ENSG00000242574.8_3	HLA‐DMB	Up	3.07339
ENSG00000213699.8_3	SLC35F6	Up	3.00963
ENSG00000131747.14_2	TOP2A	Up	2.90592
ENSG00000265972.5_2	TXNIP	Down	15.27070
ENSG00000130766.4_2	SESN2	Down	7.03423
ENSG00000113739.10_2	STC2	Down	6.37436
ENSG00000204389.9_3	HSPA1A	Down	5.93096
ENSG00000166670.9_2	MMP10	Down	5.80799
ENSG00000128965.11_2	CHAC1	Down	5.75692
ENSG00000132002.7_4	DNAJB1	Down	5.71947
ENSG00000284292.1_2	AC004922.1	Down	5.63328
ENSG00000162772.16_3	ATF3	Down	5.53635
ENSG00000139112.10_3	GABARAPL1	Down	5.40852

### 3.4 GO analysis

**FIGURE 3:**
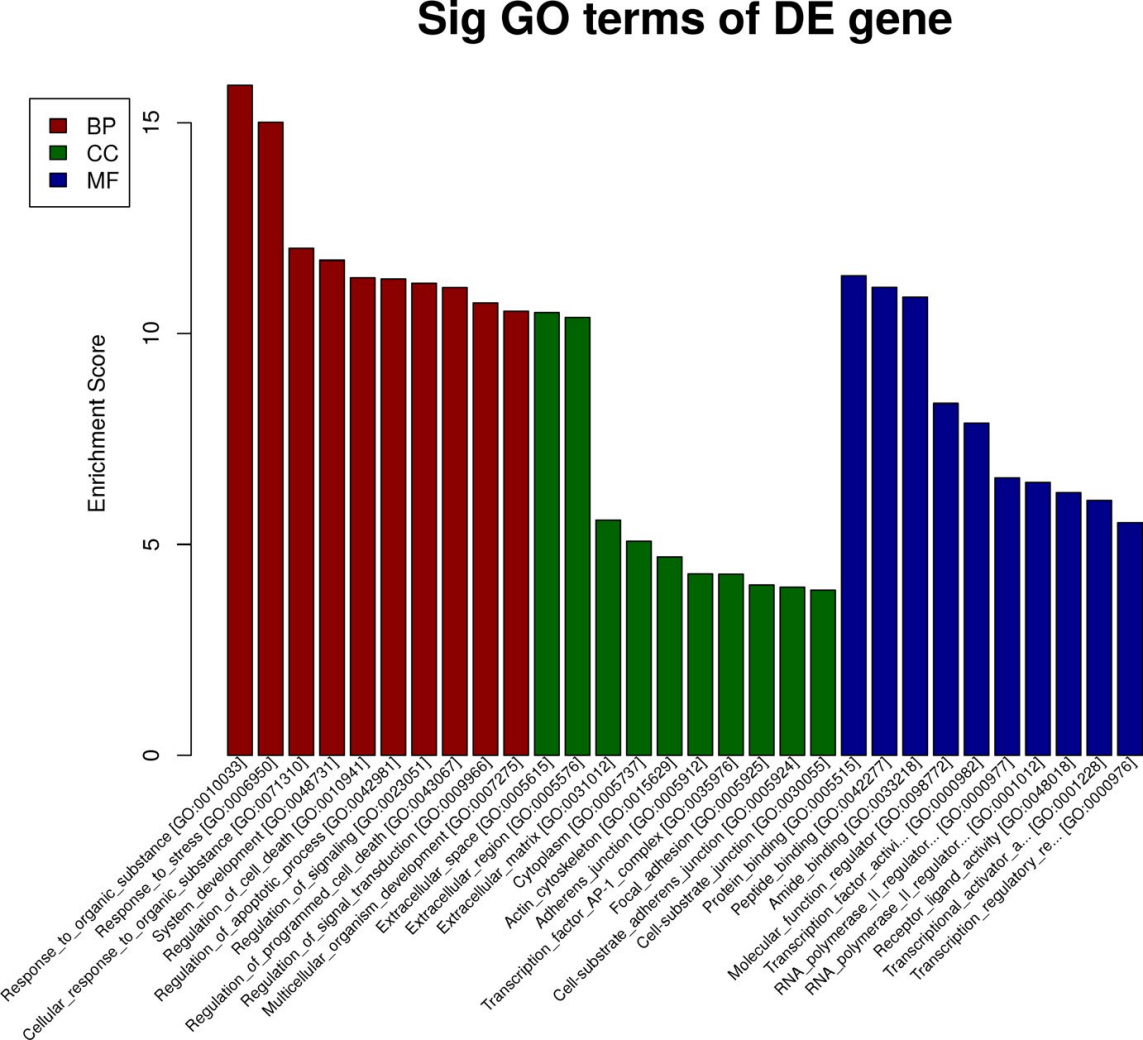
GO analysis of downregulated mRNAs‐related UCA1. The genes corresponding to the downregulated mRNAs involved in biological processes, such as response to organic substance, response to stress, cellular response to organic substance, system development, regulation of cell death, and so on. Some genes involved in cellular components, such as extracellular space, extracellular region, extracellular matrix, cytoplasm, actin cytoskeleton, and so on. Some different genes involved in molecular functions, such as protein binding, peptide binding, amide binding, molecular function regulator, transcription factor activity, RNA polymerase II proximal promoter sequence‐specific DNA binding, and so on

The genes corresponding to the downregulated mRNAs were involved in biological processes, such as response to organic substance, response to stress, cellular response to organic substance, system development, regulation of cell death, and so on. Some genes were involved in cellular components, such as extracellular space, extracellular region, extracellular matrix, cytoplasm, actin cytoskeleton, and so on. Some different genes were involved in molecular functions, such as protein binding, peptide binding, amide binding, molecular function regulator, transcription factor activity, RNA polymerase II proximal promoter sequence‐specific DNA binding, and so on (shown in Figure 3).

**FIGURE 4:**
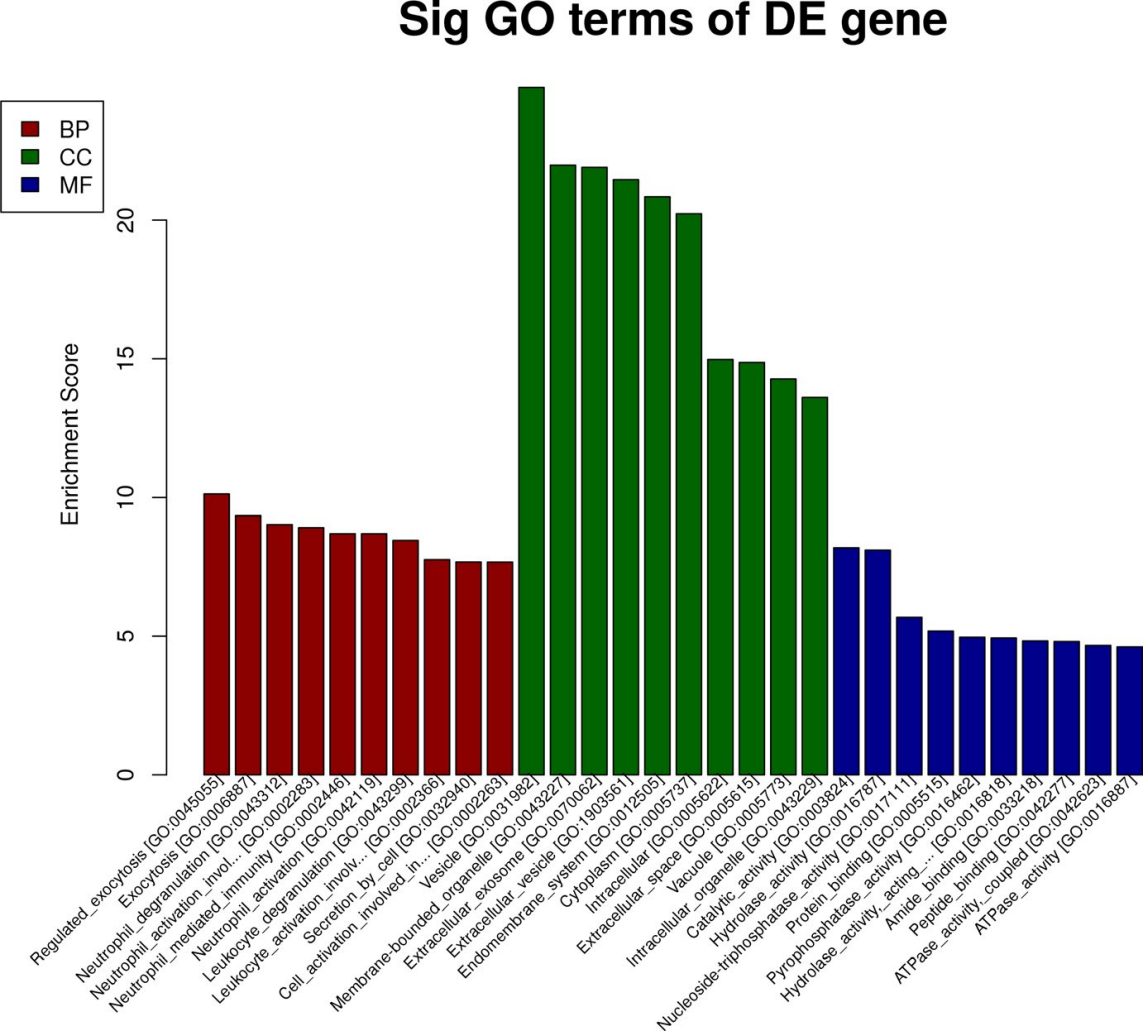
GO analysis of upregulated mRNAs‐related UCA1. The genes corresponding to the upregulated mRNAs involved in biological processes, such as regulated exocytosis, exocytosis, neutrophil degranulation, neutrophil activation involved in immune response, neutrophil mediated immunity, and so on. Some genes involved in cellular components, such as vesicle, membrane‐bounded organelle, extracellular exosome, extracellular vesicle, endomembrane system, and so on. Some different genes involved in molecular functions, such as catalytic activity, hydrolase activity, nucleoside‐triphosphatase activity, protein binding, pyrophosphatase activity, and so on

The genes corresponding to the upregulated mRNAs were involved in biological processes, such as regulated exocytosis, exocytosis, neutrophil degranulation, neutrophil activation involved in immune response, neutrophil mediated immunity, and so on. Some genes were involved in cellular components, such as vesicle, membrane‐bounded organelle, extracellular exosome, extracellular vesicle, endomembrane system, and so on. Some different genes were involved in molecular functions, such as catalytic activity, hydrolase activity, nucleoside‐triphosphatase activity, protein binding, pyrophosphatase activity, and so on (shown in Figure 4).

### 3.5 Pathway analysis

**FIGURE 5:**
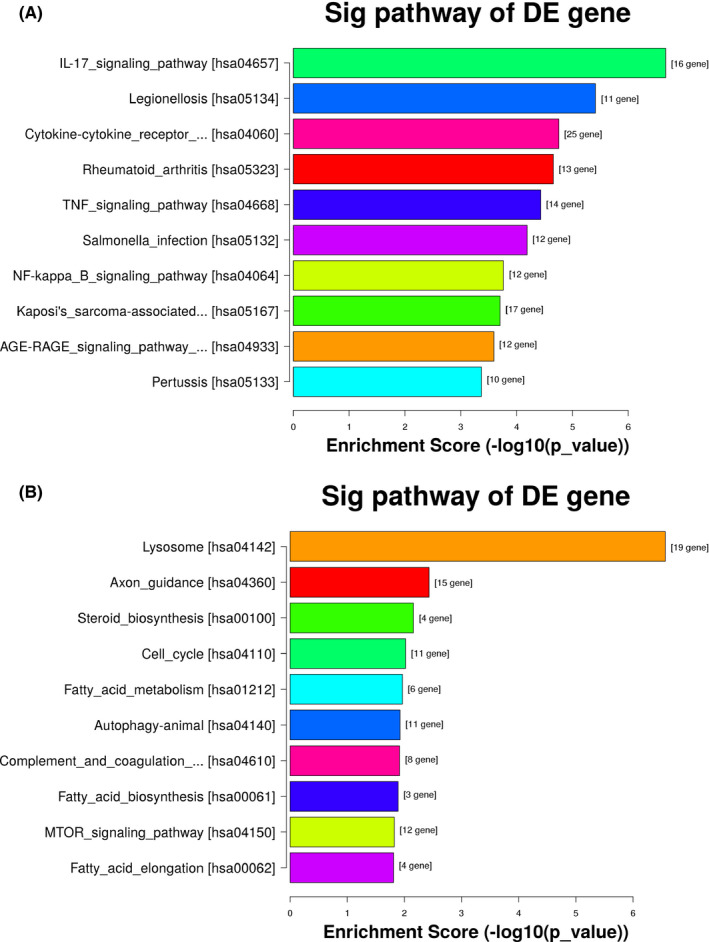
Pathway analysis. A, These different genes involved into 55 downregulated pathways were identified, including IL‐17 signaling pathway, legionellosis, cytokine‐cytokine receptor interaction, rheumatoid arthritis, TNF signaling pathway, and so on. B, These different genes involved into 22 upregulated pathways were identified, including lysosome, axon guidance, steroid biosynthesis, cell cycle, fatty acid metabolism, and so on

These different genes involved into 22 upregulated pathways were identified, including lysosome, axon guidance, steroid biosynthesis, cell cycle, fatty acid metabolism, and so on. These different genes involved into 55 down‐ regulated pathways were identified, including IL‐17 signaling pathway, legionellosis, cytokine‐cytokine receptor interaction, rheumatoid arthritis, TNF signaling pathway, and so on (shown in Figure 5A,B).

### 3.6 Real‐time quantitative PCR validation

**FIGURE 6:**
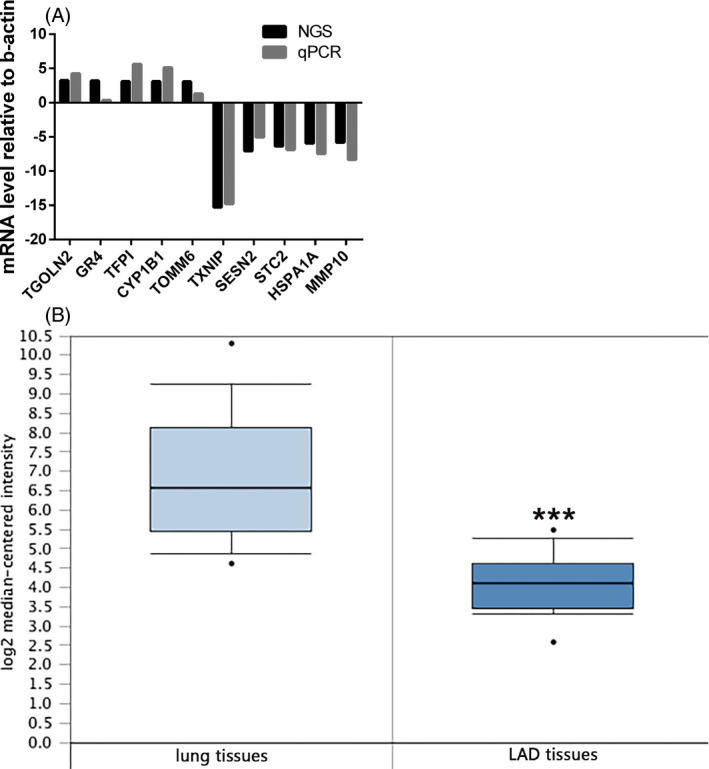
qPCR validation result. A, TGOLN2, GR4, TFPI, CYP1B1, TOMM6, TXNIP, SESN2, STC2, HSPA1A, and MMP10 and found that qPCR of most candidate mRNAs was relative to NGS. The NGS showed that the TXNIP mRNA expression level was the most significantly downexpressed changed mRNA of these mRNAs (fold change = 15.27070, *P* = .005815). B, TXNIP mRNA expression level of LAD tissues was clearly lower than the adjacent tissues (fold change = −6.623, *t *= −6.220, *P* = 1.53E−6). ***P < .001

**FIGURE 7:**
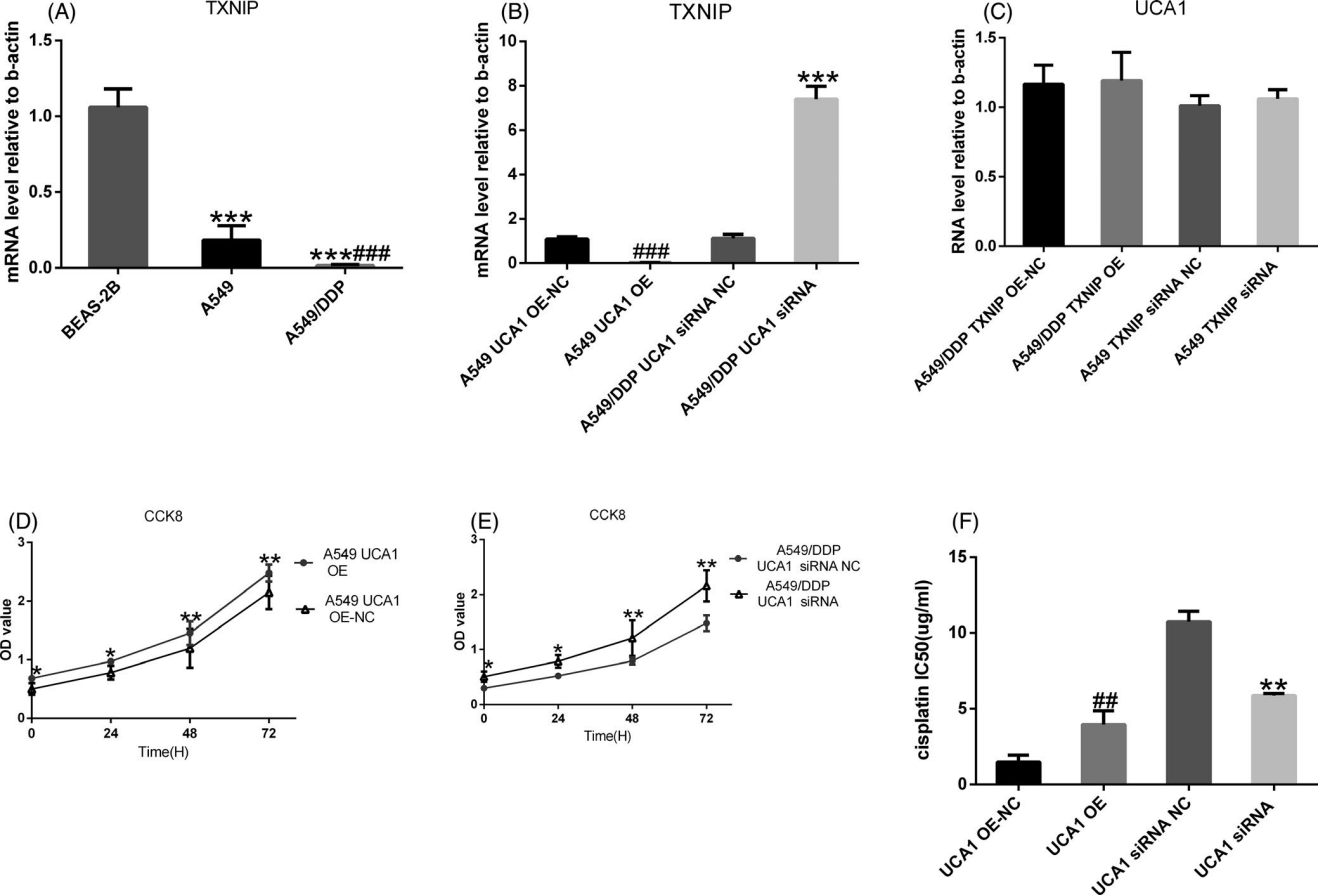
UCA1 promotes cisplatin resistance in lung adenocarcinoma by downregulating TXNIP expression. A, Compared to the BEAS‐2B, TXNIP mRNA expression level cut down in A549 and A549/DDP cell (*t* = 9.863, *P* = .0006 and *t* = 14.85, *P* = .0001) and that of A549/DDP cell was lower than A549 cell (*t* = 3.092, *P* = .0365). B, After knocking down UCA1, TXNIP mRNA was significantly heightened (*t* = 18.12, *P* < .0001). After overexpression of UCA1, TXNIP mRNA decreased significantly (*t* = 16.84, *P* < .0001). C, After knocking down and overexpression of TXNIP, UCA1 RNA expression level did not clearly change (*t* = 0.1879, *P* = .8601 and *t* = 0.8982, *P* = .4199). D, Proliferation ability of A549 UCA1 OE cell in 0 h (*P* < .05), 24 h (*P* < .05), 48 h (*P* < .01), and 72 h (*P* < .01) was higher than that of A549 UCA1 OE NC cell in 0, 24, 48, 72 h. (E) The proliferation ability of A549/DDP UCA1 siRNA cell in 0 h (*P* < .05), 24 h (*P* < .05), 48 h (*P* < .01), and 72 h (*P* < .01) was lower than that of A549/DDP UCA1 siRNA NC cell in 0, 24, 48, and 72 h. (F) After overexpression of UCA1, IC50 lifted significantly (*t* = 4.175, *P* = .0140) and IC50 cut down clearly (*t* = 11.81, *P* = .0003) after knocking down UCA1. *P < .05, **P < .01, ***P < .001. #*P* < .05, ##*P* < .01, ###*P* < .001

According to fold difference, gene locus, nearby encoding gene, and so on, we initially identified several interesting candidate mRNAs (including TGOLN2, GR4, TFPI, CYP1B1, TOMM6, TXNIP, SESN2, STC2, HSPA1A, and MMP10) and found that qPCR results of most candidate mRNAs were relative to NGS; see Figure 6A. The NGS shown that the TXNIP mRNA expression level was the most significantly downexpressed changed mRNA of these mRNAs (fold change = 15.27070, *P* = .005815).TXNIP mRNA expression level of LAD tissues was distinctly lower than the adjacent tissues (fold change = −6.623, *t *= −6.220, *P* = 1.53E−6); see Figure 6B. QPCR shown that UCA1 expression level in cisplatin insensitive group was markedly higher than that in cisplatin‐sensitive group (*t* = 9.019, *P* < .0001), while TXNIP mRNA expression level in cisplatin insensitive group was clearly lower than that in cisplatin‐sensitive group (*t* = 8.287, *P* < .0001); see Figure 6C,D. Compared to the BEAS‐2B, TXNIP mRNA expression level markedly lowered in A549 and A549/DDP cell (*t* = 9.863, *P* = .0006 and *t* = 14.85, *P* = .0001) and that of A549/DDP cell was lower than A549 cell (*t* = 3.092, *P* = .0365); see Figure 7A. This result indicated that TXNIP had a significant negative correlation with UCA1 expression, indicating that this gene may be a target gene downstream of UCA1.

### 3.7 UCA1 promotes cisplatin resistance in lung adenocarcinoma by downregulating TXNIP expression

According to Figure 7, after overexpression of UCA1, TXNIP mRNA visibly decreased (*t* = 16.84, *P* < .0001); see Figure 7B, and proliferation ability of A549 UCA1 OE cell in 0 hour (*P* < .05), 24 hours (*P* < .05), 48 hours (*P* < .01), and 72 hours (*P* < .01) was higher than that of A549 UCA1 OE NC cell in 0, 24, 48, and 72 hours (see Figure 7D). IC50 markedly elevated (*t* = 4.175, *P* = .0140, Figure 7F). After knocking down UCA1, TXNIP mRNA was significantly lifted (*t* = 18.12, *P* < .0001); see Figure 7B, and proliferation ability of A549/DDP UCA1 siRNA cell in 0 hour (*P* < .05), 24 hours (*P* < .05), 48 hours (*P* < .01), 72 hours (*P* < .01) was lower than that of A549/DDP UCA1 siRNA NC cell in 0, 24, 48, 72 hours (see Figure 7E), and IC50 reduced clearly (*t* = 11.81, *P* = .0003, Figure 7F). After knocking down and overexpression of TXNIP, UCA1 RNA expression level did not visibly change (*t* = 0.1879, *P* = .8601 and *t* = 0.8982, *P* = .4199, Figure 7C).

### 3.8 Interaction prediction between TXNIP and protein

**FIGURE 8:**
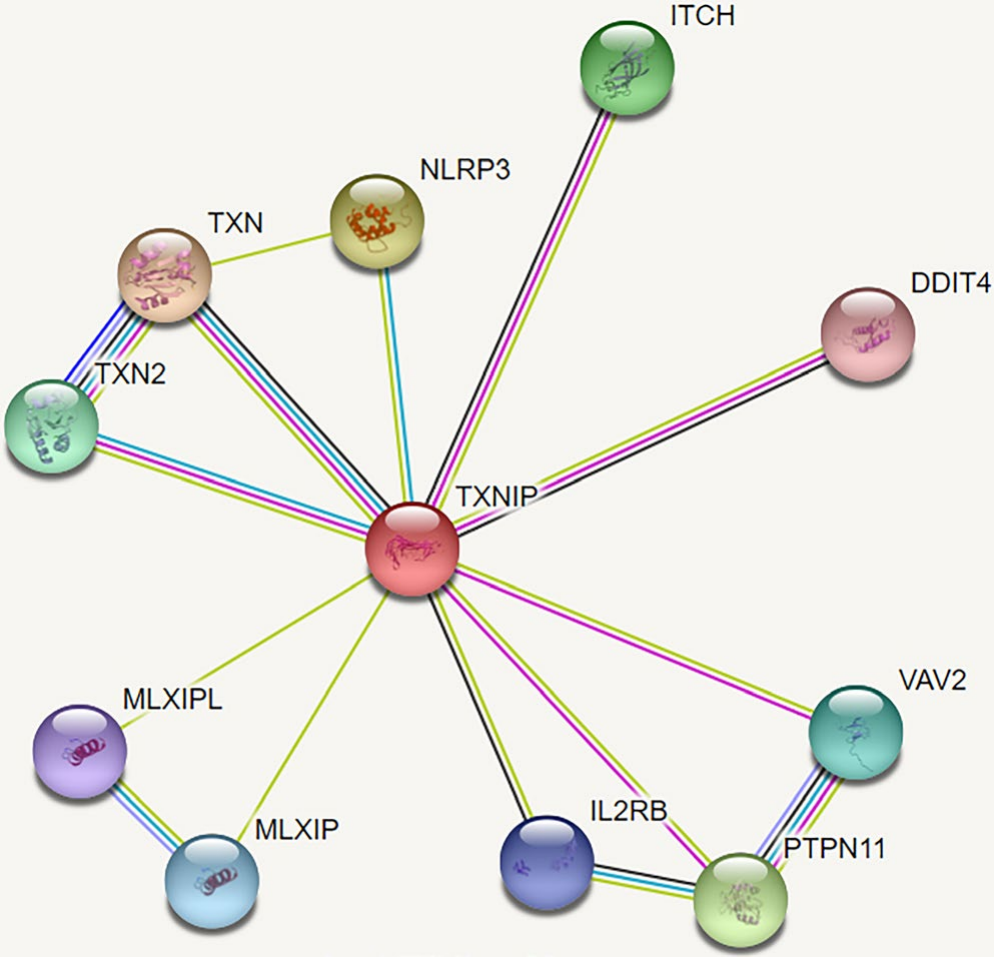
Interaction prediction between TXNIP and protein. The analysis results from https://string‐db.org/ show that TXNIP could interact with multiple proteins such as TXN, DDIT4, and NLRP3

The analysis results from https://string‐db.org/ shows that TXNIP could interact with multiple proteins such as TXN, DDIT4, and NLRP3 (Figure 8). These results showed that TXNIP might play a key role in cisplatin resistance of LAD by interacting with some proteins.

## 4 DISCUSSION

It was extremely complex about the mechanism of cisplatin resistance and involved into multiple genes, proteins, and several pathways. It was currently believed to be achieved mainly through these mechanisms,^18^, ^19^, ^20^, ^21^, ^22^ including altering intracellular drugs transport, reducing interferes of drug ability with some mechanisms, affecting DNA damage relative repairing gene, and resulting in genetic and epigenetic changes of the main pathways.

The results showed that 647 upregulated mRNAs and 633 downregulated mRNAs differentially expressed (*P* < .05) between A549 OE and A549 OE NC group and hinted that UCA1 might affect these different genes expression, and UCA1 was a very important lncRNA gene in cisplatin resistance of LAD. We initially identified several interesting different mRNA genes (including TGOLN2, GR4, TFPI, CYP1B1, TOMM6, TXNIP, SESN2, STC2, HSPA1A, and MMP10) and found that qPCR of most candidate mRNAs was relative to gene chip.

In order to obtain insights into UCA1 target gene function, GO analysis and KEGG pathway annotation were applied to the UCA1 target gene pool. GO analysis revealed that the genes corresponding to the upregulated and downregulated mRNAs all involved in biological processes, cellular components, and molecular functions. KEGG annotation showed that these different genes involved into 22 upregulated pathways were identified, including lysosome, axon guidance, steroid biosynthesis, cell cycle, fatty acid metabolism, and so on. These different genes involved into 55 downregulated pathways were identified, including IL‐17 signaling pathway, legionellosis, cytokine‐cytokine receptor interaction, rheumatoid arthritis, TNF signaling pathway, and so on. These pathways might be important downstream signaling pathways of UCA1 and play important roles in cisplatin resistance of LAD.

We found that TXNIP mRNA expression level was the most visibly downexpressed changed of these different mRNAs, and TXNIP mRNA expression level of LAD tissues was clearly lower than the adjacent tissues. UCA1 expression level in cisplatin insensitive group was markedly higher than that in cisplatin‐sensitive group, while TXNIP mRNA expression level in cisplatin insensitive group was clearly lower than that in cisplatin‐sensitive group. Compared to the BEAS‐2B, TXNIP mRNA expression level visibly decreased in A549 and A549/DDP cell, and that of A549/DDP cell was lower than A549 cell. After UCA1 overexpression, TXNIP had been proven to be a tumor suppressor gene.^23^, ^24^, ^25^ We furtherly found that TXNIP mRNA reduced obviously and proliferation ability, IC50 of A549 elevated significantly. After knocking down UCA1, TXNIP mRNA was significantly increased, and proliferation ability and IC50 of A549/DDP clearly reduced. After knocking down and overexpression of TXNIP, UCA1 RNA expression level did not distinctly change. These results showed that TXNIP was a downstream target molecule of UCA1 and UCA1/TXNIP axis might affect cisplatin resistance in LAD. PPI analysis result showed that TXNIP could interact with multiple proteins such as TXN, DDIT4, and NLRP3. Among them, TXN and DDIT4 had been confirmed by literature reports,^26^, ^27^ while NLRP3, MLXIPL, MLXIP, IL2RB, ITCH, etc have not been experimentally confirmed and need further study.

## 5 CONCLUSIONS

Our study ascertained UCA1 could regulate a lot of downstream pathways and target genes. UCA1 promoted cisplatin resistance by downregulating TXNIP expression in LAD, and TXNIP could interact with multiple proteins. So, UCA1/TXNIP axis might affect cisplatin resistance in LAD.

## CONFLICT OF INTEREST

The authors declare that they have no competing interests.

## ETHICAL APPROVAL

Written informed consent was obtained from all patients. The study was approved by the Ethical Committee of the First Affiliated Hospital of Wenzhou Medical University.

## References

[1] Pignon J‐P , Tribodet H , Scagliotti GV , et al. Lung adjuvant cisplatin evaluation: a pooled analysis by the LACE Collaborative Group. J Clin Oncol. 2008;26:3552‐3559. 18506026 10.1200/JCO.2007.13.9030

[2] Zarogoulidis K , Zarogoulidis P , Darwiche K , et al. Treatment of non‐small cell lung cancer (NSCLC). J Thorac Dis. 2013;5(Suppl 4):S389‐396. 24102012 10.3978/j.issn.2072-1439.2013.07.10 PMC3791496

[3] Bunn PA Jr , Kelly K . New combinations in the treatment of lung cancer: a time for optimism. Chest. 2000;117:138S‐143S. 10777469 10.1378/chest.117.4_suppl_1.138s

[4] Spiro SG , Silvestri GA . One hundred years of lung cancer. Am J Respir Crit Care Med. 2005;172:523‐529. 15961694 10.1164/rccm.200504-531OE

[5] Choi MK , Kim DD . Platinum transporters and drug resistance. Arch Pharm Res. 2006;29:1067‐1073. 17225452 10.1007/BF02969293

[6] Wang XS , Zhang Z , Wang HC , et al. Rapid identification of UCA1 as a very sensitive and specific unique marker for human bladder carcinoma. Clin Cancer Res. 2006;12:4851‐4858. 16914571 10.1158/1078-0432.CCR-06-0134

[7] Nie W , Ge H‐J , Yang X‐Q , et al. LncRNA‐UCA1 exerts oncogenic functions in non‐small cell lung cancer by targeting miR‐193a‐3p. Cancer Lett. 2016;371:99‐106. 26655272 10.1016/j.canlet.2015.11.024

[8] Wang HM , Lu JH , Chen WY , Gu AQ . Upregulated lncRNA‐UCA1 contributes to progression of lung cancer and is closely related to clinical diagnosis as a predictive biomarker in plasma. Int J Clin Exp Med. 2015;8:11824‐11830. 26380024 PMC4565407

[9] Fan YU , Shen B , Tan M , et al. Long non‐coding RNA UCA1 increases chemoresistance of bladder cancer cells by regulating Wnt signaling. FEBS J. 2014;281:1750‐1758. 24495014 10.1111/febs.12737

[10] Wang F , Zhou J , Xie X , et al. Involvement of SRPK1 in cisplatin resistance related to long non‐coding RNA UCA1 in human ovarian cancer cells. Neoplasma. 2015;62:432‐438. 25967360 10.4149/neo_2015_051

[11] Dimitriadou E , Noutsopoulos D , Markopoulos G , et al. Abnormal DLK1/MEG3 imprinting correlates with decreased HERV‐K methylation after assisted reproduction and preimplantation genetic diagnosis. Stress. 2013;16:689‐697. 23786541 10.3109/10253890.2013.817554

[12] Pertea M , Pertea GM , Antonescu CM , Chang TC , Mendell JT , Salzberg SL . StringTie enables improved reconstruction of a transcriptome from RNA‐seq reads. Nat Biotechnol. 2015;33:290‐295. 25690850 10.1038/nbt.3122 PMC4643835

[13] Pertea M , Kim D , Pertea GM , Leek JT , Salzberg SL . Transcript‐level expression analysis of RNA‐seq experiments with HISAT, StringTie and Ballgown. Nat Protoc. 2016;11:1650‐1667. 27560171 10.1038/nprot.2016.095 PMC5032908

[14] Mortazavi A , Williams BA , McCue K , Schaeffer L , Wold B . Mapping and quantifying mammalian transcriptomes by RNA‐Seq. Nat Methods. 2008;5:621‐628. 18516045 10.1038/nmeth.1226 PMC13303166

[15] Robinson JT , Thorvaldsdóttir H , Winckler W , et al. Integrative genomics viewer. Nat Biotechnol. 2011;29:24‐26. 21221095 10.1038/nbt.1754 PMC3346182

[16] Wang L , Park HJ , Dasari S , Wang S , Kocher JP , Li W . CPAT: Coding‐Potential Assessment Tool using an alignment‐free logistic regression model. Nucleic Acids Res. 2013;41:e74. 23335781 10.1093/nar/gkt006 PMC3616698

[17] Ren S , Peng Z , Mao J‐H , et al. RNA‐seq analysis of prostate cancer in the Chinese population identifies recurrent gene fusions, cancer‐associated long noncoding RNAs and aberrant alternative splicings. Cell Res. 2012;22:806‐821. 22349460 10.1038/cr.2012.30 PMC3343650

[18] Ohmichi M , Hayakawa J , Tasaka K , Kurachi H , Murata Y . Mechanisms of platinum drug resistance. Trends Pharmacol Sci. 2005;26:113‐116. 15749154 10.1016/j.tips.2005.01.002

[19] Seve P , Dumontet C . Chemoresistance in non‐small cell lung cancer. Curr Med Chem Anticancer Agents. 2005;5:73‐88. 15720263 10.2174/1568011053352604

[20] Wu C , Wangpaichitr M , Feun L , et al. Overcoming cisplatin resistance by mTOR inhibitor in lung cancer. Mol Cancer. 2005;4:25. 16033649 10.1186/1476-4598-4-25 PMC1181826

[21] Martin LP , Hamilton TC , Schilder RJ . Platinum resistance: the role of DNA repair pathways. Clin Can Res. 2008;14:1291‐1295. 10.1158/1078-0432.CCR-07-2238 18316546

[22] Wangpaichitr M , Wu C , You M , et al. Inhibition of mTOR restores cisplatin sensitivity through down‐regulation of growth and anti‐apoptotic proteins. Eur J Pharmacol. 2008;591:124‐127. 18585380 10.1016/j.ejphar.2008.06.028 PMC2744337

[23] Morrison JA , Pike LA , Sams SB , et al. Thioredoxin interacting protein (TXNIP) is a novel tumor suppressor in thyroid cancer. Mol Cancer. 2014;13:62. 24645981 10.1186/1476-4598-13-62 PMC3995095

[24] Zhang C , Wang H , Liu X , et al. Oncogenic microRNA‐411 promotes lung carcinogenesis by directly targeting suppressor genes SPRY4 and TXNIP. Oncogene. 2019;38:1892‐1904. 30390072 10.1038/s41388-018-0534-3 PMC6475890

[25] Wilde BR , Ayer DE . Interactions between Myc and MondoA transcription factors in metabolism and tumourigenesis. Br J Cancer. 2015;113:1529‐1533. 26469830 10.1038/bjc.2015.360 PMC4705882

[26] Pasternak Y , Ohana M , Biron‐Shental T , Cohen‐Hagai K , Benchetrit S , Zitman‐Gal T . Thioredoxin, thioredoxin interacting protein and transducer and activator of transcription 3 in gestational diabetes. Mol Biol Rep. 2019;47:1199‐1206. 31848914 10.1007/s11033-019-05221-8

[27] Ratushnyy AY , Rudimova YV , Buravkova LB . Alteration of hypoxia‐associated gene expression in replicatively senescent mesenchymal stromal cells under physiological oxygen level. Biochemistry Biokhimiia. 2019;84:263‐271. 31221064 10.1134/S0006297919030088

